# A training manual for event history analysis using longitudinal data

**DOI:** 10.1186/s13104-019-4544-1

**Published:** 2019-08-14

**Authors:** Philippe Bocquier, Carren Ginsburg, Mark A. Collinson

**Affiliations:** 1 Centre de Recherche en Démographie, Université Catholique de Louvain, Place Montesquieu, 1 bte L2.08.03, 1348 Louvain‐la‐Neuve, Belgium; 2 Medical Research Council/Wits Rural Public Health and Health Transitions Research Unit (Agincourt), School of Public Health, Faculty of Health Sciences, University of the Witwatersrand, Johannesburg, South Africa; 3 Department of Science and Technology/Medical Research Council, South African Population Research Infrastructure Network, Johannesburg, South Africa

**Keywords:** Longitudinal data analysis, Demographic rates, Event history analysis, Health and Demographic Surveillance System

## Abstract

**Objective:**

This research note reports on the activities of the Multi-centre Analysis of the Dynamics of Internal Migration And Health (MADIMAH) project aimed at collating and testing of a set of tools to conduct longitudinal event history analyses applied to standardised Health and Demographic Surveillance System (HDSS) datasets. The methods are illustrated using an example of longitudinal micro-data from the Agincourt HDSS, one of a number of open access datasets available through the INDEPTH iShare2 data repository. The research note documents the experience of the MADIMAH group in analysing HDSS data and demonstrates how complex analyses can be streamlined and conducted in an accessible way. These tools are aimed at aiding analysts and researchers wishing to conduct longitudinal data analysis of demographic events.

**Results:**

The methods demonstrated in this research note may successfully be applied by practitioners to longitudinal micro-data from HDSS, as well as retrospective surveys or register data. The illustrations provided are accompanied by detailed, tested computer programs, which demonstrate the full potential of longitudinal data to generate both cross-sectional and longitudinal standard descriptive estimates as well as more complex regression estimates.

## Introduction

The Multi-centre Analysis of the Dynamics of Internal Migration and Health (MADIMAH) project was conceived in 2011 to provide much-needed evidence on relationships between migration and health in sub-Saharan Africa [1]. The project recognised the potential for Health and Demographic Surveillance Systems (HDSS) data to be employed using a standardised methodology and analytical framework to generate comparative results across diverse settings. HDSS monitor all births, deaths and in- and out-migrations in a geographically-defined population, generating prospective longitudinal data with a precise temporal dimension. Employing these data to produce evidence on migration dynamics has been the focus of the MADIMAH project.

Following the experience of the MADIMAH project, the International Network for the Demographic Evaluation of Populations and their Health (INDEPTH) have facilitated the public release of HDSS data from low- and middle-income countries (LMIC) through the iSHare data repository [2]. To date there are 34 core standardised longitudinal datasets from HDSSs located in the African, Asian and Pacific Regions available in this open resource [3].

A central aim of MADIMAH has been to advance a set of tools for data management and application of event history analysis (EHA) to encourage the use of these high quality, publically available data. This initiative seeks to fill the gap in longitudinal population data available in LMIC, which are crucial to understanding population dynamics and their consequences. The objective of this research note is to document a set of EHA tools to produce reliable and comparable statistical results. The research note is accompanied by a training manual (Additional file 1) that guides the user through EHA, illustrating how to produce standard cross-sectional and longitudinal demographic rates and advanced EHA using individual-level datasets. These tools build on a previously published data management training manual [4] that was developed to guide users through a set of procedures to produce HDSS datasets in a harmonised structure.

The EHA methods illustrated in this research note and described in detail in the accompanying training manual (Additional file 1), represent a collection of tools for analysis of longitudinal HDSS data. The MADIMAH project team has collated these methods based on its experiences of conducting multi-centre analyses of migration and mortality. The methods described have been tested on and applied to more than 30 HDSS datasets. Over the past 8 years, the MADIMAH team has brought together data managers, analysts and students from HDSS centres across sub-Saharan Africa to train on and apply these techniques to HDSS data. The accompanying manual, written in an accessible language but with the necessary statistical rigour, is targeted at researchers and analysts from multidisciplinary backgrounds (including demography, public health, epidemiology and statistics) who are interested in conducting longitudinal data analysis of demographic events.

## Main text

### Methods

Traditionally, demographic estimates have been based on cross-sectional or aggregate data. These calculations of demographic rates, dominant in publications, usually involve estimating the population at mid-period of interest as well as a count of the number of events of interest over the period. For example, a death rate that is computed according to the following formula requires that the total number of deaths in a population be counted and divided by the total mid-year population:

$$ {\text{Crude death rate }} = \frac{Total \,number\,of\,deaths\,\,in\,a\,given\,year }{total\,\,mid - year\,\,population} = \frac{{D_{{\left( {t, t + n} \right)}} }}{{\left( {P_{t} + P_{t + n} } \right)/2}} $$

This is often estimated based on the population at the start of the year added to the population at the end of the year, divided by two. These methods suffer from inaccuracies regarding the handling of events such as migration, and cannot easily deal with the issue of censoring [5]. Also, with such aggregates, it is not straightforward to obtain cohort measures of probabilities except through the application of formulas that convert rates to probabilities using approximate average person-years lived in the age interval [5]. The event history analyses (EHA) approach allows for the computation of exact person-years, and can successfully handle right- and left-censored data to produce estimates based on both calendar years and age groups. In addition to the computation of descriptive indicators (such as birth, death, in- and out-migration rates and probabilities), longitudinal data sources may be effectively utilised for more sophisticated EHA [6].

The analytic methods presented in this research note are illustrated using HDSS data but can also be applied to register or retrospective survey data. We use the Agincourt HDSS core micro dataset available for download through the INDEPTH iSHARE2 data repository [3]. The analytical dataset was extended to include data on causes of death (CoD) to exemplify the analysis of competing risks in the last section of the attached manual (Additional file 1), and these data are available upon reasonable request to the Agincourt HDSS site (https://www.agincourt.co.za/). The Agincourt HDSS was established in 1992 and is located in the rural north-east of South Africa. The surveillance population currently comprises over 90,000 individuals living in 11,500 households [7].

The core micro dataset, or core residency file, is a standardised file format containing the key events for each individual in the surveillance population with each event being documented as a single record. This type of dataset considers events that change the residency status of the individual (such as: enumeration, birth, death, in-migration, out-migration and end of observation). For each event, a corresponding event date is captured (see the MADIMAH team’s first manual of data management for more detail [4]).

The results below illustrate with the Agincourt HDSS micro data how to use standard commands available in most statistical software packages. Our illustrations and corresponding code in the attached manual (Additional file 1) uses a suite of Stata^®^ version 15 commands. We highlight below new techniques such as the cumulative incidence function for competing risks such a causes of death or the reverse-time for the computation of in-migration rates. The results illustrate how a set of techniques applied to longitudinal HDSS data can be integrated to avoid unnecessary division between descriptive and more complex analyses.

### Results

**Fig. 1:**
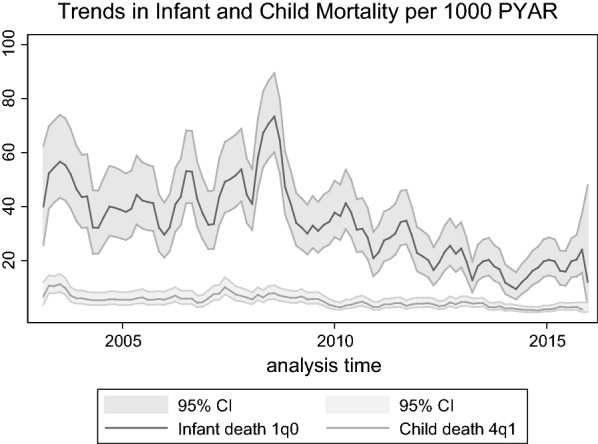
Infant and child death hazard functions by calendar time (source: Agincourt HDSS 2003–2015)

The foundation statistic in EHA is the hazard rate by age [5]. This rate represents the risk in a given short age interval of experiencing the event. It is expressed as an annualised probability, i.e. a number of events per 1000 person-years. The hazard curve is usually represented by age, sometimes for a specific calendar period. However, the hazard function need not be represented by age. Using the same data, one can represent the hazard function by calendar time, for the whole population but more often for a specific age group. Figure 1 is an illustration of hazard curves, with infant and child death hazards from 1 January 2013 to 31 December 2015. One can clearly see a drop in infant mortality from 2009 (antiretroviral treatment were largely made available free-of-charge from 2008 in the study area). The attached manual (Additional file 1) gives time-scale recommendations for smoothing hazard rates in a meaningful way in relation to data collection precision in dates and proportion of events.

The above figure is for data exploration and for communication (to show levels and changes in trends) but may also be presented in tables. Two different indicators are used in the literature: rates and probabilities. Rates (_n_m_x_) most closely correspond to hazard rates except that they are usually defined for conventional age groups [5]. They are defined as the number of events over the total person-years accounted for in a given age interval, as exemplified in Table 1. The attached manual (Additional file 1) shows how to produce such a table for each calendar periods to identify mortality, migration or fertility trends, e.g. by 5-year age group and 5-year period.

**Table 1:** Death rates and survival probability by age group for males. Source: Agincourt HDSS 2003–2015

Death rates by age group for males						Survival probability by age group for males					
Age	Person-time	Failures	Rate	95% Conf. interval		Time	beg. total	Fail	Survivor function	95% Conf. interval	
				Min	Max					Min	Max
~	~	~	~	~	~	0	0	0	1.0000	~	~
(0–1]	13,832.24	449	32.46	29.59	35.61	1	13819	449	0.9681	0.9650	0.9708
(1–5]	54,138.84	285	5.26	4.69	5.91	5	13,307	285	0.9480	0.9442	0.9516
(5–10]	65,132.29	98	1.50	1.23	1.83	10	12,788	98	0.9409	0.9369	0.9447
(10–15]	64,674.41	75	1.16	0.92	1.45	15	13,474	75	0.9355	0.9313	0.9394
(15–20]	67,388.26	82	1.22	0.98	1.51	20	13,446	82	0.9298	0.9254	0.9339
(20–25]	64,555.91	184	2.85	2.47	3.29	25	12,204	184	0.9166	0.9119	0.9210
(25–30]	54,168.97	384	7.09	6.41	7.83	30	9551	384	0.8841	0.8785	0.8894
(30–35]	41,721.90	530	12.70	11.67	13.83	35	7457	530	0.8293	0.8224	0.8360
(35–40]	31,826.70	530	16.65	15.29	18.13	40	5629	530	0.7632	0.7548	0.7713
(40–45]	24,481.75	542	22.14	20.35	24.08	45	4388	542	0.6830	0.6731	0.6927
(45–50]	18,611.63	400	21.49	19.49	23.70	50	3300	400	0.6131	0.6021	0.6240
(50–55]	14,558.26	366	25.14	22.69	27.85	55	2607	366	0.5406	0.5286	0.5524
(55–60]	11,412.78	308	26.99	24.14	30.18	60	1950	308	0.4727	0.4600	0.4852
(60–65]	8387.65	342	40.77	36.67	45.33	65	1466	342	0.3857	0.3725	0.3990
(65–70]	6064.58	268	44.19	39.20	49.81	70	1002	268	0.3085	0.2950	0.3220
(70–75]	4421.76	264	59.70	52.92	67.36	75	768	264	0.2284	0.2155	0.2414
(75–80]	2989.68	220	73.59	64.48	83.98	80	466	220	0.1566	0.1450	0.1687
(80–85]	1921.65	197	102.52	89.16	117.88	85	329	197	0.0938	0.0843	0.1039
> 85	1642.42	240	146.13	128.76	165.83	120	1	240	~	~	~

The other way to represent event intensity is through the survivor function that represents the probability to survive until a given age (_n_q_x_) for a synthetic cohort, i.e. a cohort of individuals that would have been subjected over their lifetimes to the conditions prevailing over the observed period (see Table 1). Both the death rates (_n_m_x_) and survival probabilities (_n_q_x_) may be computed from the same data without resorting to conversion formulas as necessary with aggregates. The distribution of events by age interval is the same for _n_m_x_ and _n_q_x_. Aggregates (column 8) are not accurate since, as noted in the Stata output, the “survivor function is calculated over full data and evaluated at indicated times; it is not calculated from aggregates.” More reliable are the person-years displayed in column 2. Common summary cohort measures, such as life expectancy or median age at death are derived from the probabilities.

**Fig. 2:**
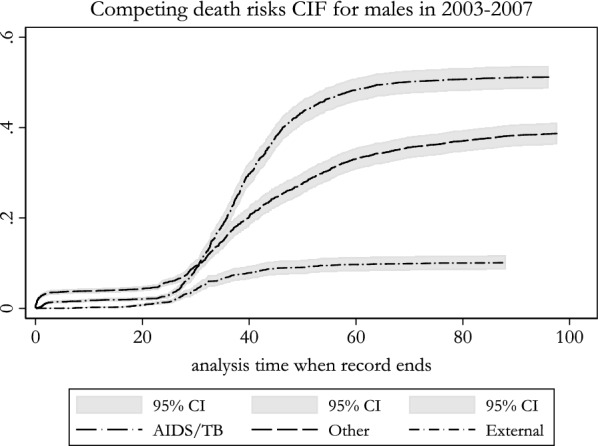
Cumulative incidence function (CIF) for three large causes of death for males (source: Agincourt HDSS 2003–2007, indeterminate causes of death excluded)

Another useful synthetic cohort descriptive tool is the cumulative incidence function (CIF) [8] that has not so far been presented in published manuals. We recommend this over the cumulative hazard function also known as the Nelson-Aalen function (NAF) to analyse competing events such as causes of death, which is based on the assumption of independence between competing events that doesn’t always hold. The advantage of the CIF over the NAF is that the sum of CIF for each competing event is equal to the Kaplan–Meier failure function, unlike the NAF whose scale has no clear interpretation (it frequently exceeds the value 1). However the NAF is still useful for repeatable events (competing or not) since the CIF does not handle repeatable events. Figure 2 presents the CIF for large categories of death. AIDS/TB represents about half of the mortality intensity in the 2003–2007 period.

An original contribution that the MADIMAH team has streamlined is the detailed procedure to analyse in-migration [9, 10]. This is a special case in event history analysis that involves reversing analysis time to compute rates using destination population at risk instead of the origin population at risk (as done for out-migration analysis).

The full potential of longitudinal data relates not only to the ability to produce standard descriptive estimates as we have seen above, but also to the ability to produce more complex regression estimates. The well-known Cox model (semi-parametric proportional hazard model is its full name) and the less known Fine and Gray model for non-independent competing risks [11] can easily be implemented using the same micro data that we used to produce rates and probabilities. The MADIMAH team has successfully applied these methods to analyses of determinants and outcomes of demographic processes, to produce results that are comparable across diverse settings [12, 13].

## Limitations

The computer programs and analyses outlined in this research note are flexible and can be applied to renewable or non-renewable events, competing risks or non-competing risks. However, consideration should be given as to the time-precision of the data, the precision of recorded dates for data collection (e.g., days) should always be higher than the unit of time of analysis (e.g., years). The manual (Additional file 1) has been designed for Stata users and the provided computer programs would require adaptation for use in other statistical software packages. The manual follows the previously published “Manual of event history data management using HDSS data” [4], which outlines the steps to structure the data into the required format for EHA.

## Supplementary information

**Additional file 1.** Manual of event history data analysis using longitudinal data.

## Data Availability

The Agincourt micro data analysed during the current study is available in the INDEPTH iShare2 repository, (http://www.indepth-ishare.org/index.php/home). South Africa-Agincourt INDEPTH Core Dataset 1993–2015 (Release 2017) DDI.INDEPTH.ZA011.CMD2015.v1. Data on causes are death are available from the Agincourt HDSS on reasonable request.

## Abbreviations

CIF: cumulative incidence function
CoD: cause of death
EHA: event history analysis
HDSS: Health and Demographic Surveillance System
INDEPTH: International Network for the Demographic Evaluation of Populations and their Health
LMIC: low- and middle-income countries
NAF: Nelson-Aalen function
