# Supplementary material for: A training manual for event history analysis using longitudinal data
Source: BMC Res Notes. 2019 Aug 14;12:506. doi: 10.1186/s13104-019-4544-1 (PMC6694584; doi:10.1186/s13104-019-4544-1)
Supplement: Supplementary file 1 — Additional file 1. Manual of event history data analysis using longitudinal data. [file 13104_2019_4544_MOESM_ESM.docx]

Manual of
Event History Data Analysis
using Longitudinal Data

Philippe Bocquier and Carren Ginsburg

Foreword

The Migration, Urbanisation and Health Working Group (MUHWG), hosted by the International Network for the Demographic Evaluation of Populations and Their Health (INDEPTH), has advised on a longitudinal data format based on an event history analysis (EHA) approach used in the Multi-centre Analysis of the Dynamics of Internal Migration and Health (MADIMAH) project. This format is the basis of the micro-data specification that has now become a standard for all INDEPTH member Health and Demographic Surveillance Systems (HDSS). This is a flexible format that lends itself to the calculation of basic demographic rates, and it is also suitable for more thorough EHA. The basic standard – also named core residency file – can be easily expanded through the addition of event attributes or other status events, e.g. educational attainment, employment status, anthropometry measurements, etc.

A previous Manual of Event History Data Management presented the longitudinal data management steps, commencing with the creation of the core residency file through to producing the most sophisticated event history file. The present Manual of Event History Data Analysis is a continuation oriented towards basic analysis of longitudinal data.

Following a glossary and a general introduction to EHA, the manual is structured as follows:

- Section 2 is about calculating the basic hazard rates by age or by calendar time that form the basis for all other estimates.
- Section 3 demonstrates how to compute basic rates by period from a core residency file: this produces an annual series of out-migration, in-migration, mortality, and fertility rates by age group and sex, as may be generated from cross-sectional, register or panel data.
- Section 4 illustrates how to conduct descriptive longitudinal analysis of non-renewable events such as death. The issue of competing risks is also addressed through cause-of-death analysis.
- Section 5 illustrates how to conduct descriptive longitudinal analysis of renewable events such as migration or child delivery, and tackles the special case of in-migration analysis, which uses a reverse-time scale.
- Section 6 is a brief overview of regression techniques for longitudinal data using the semi-parametric proportional hazard Cox model and Fine and Gray model.

Acknowledgement

We would like to acknowledge scientific contributions of numerous attendees of data analysis workshops organised between 2013 and 2016 by the INDEPTH Multi-centre Analysis of the Dynamics of Internal Migration And Health (MADIMAH) group including Pr. Mark A. Collinson, Dr. Donatien Béguy, and Dr. Sulaimon Afolabi. The MADIMAH project has received funds from the Swedish International Development Agency (Sida: 2012-000379). The research has received a joint financial support from the National Research Foundation, South Africa, and the Wallonia‐Brussels Federation of Belgium (Grant No: 95284). It received support from another Wallonia‐Brussels Federation of Belgium (FNRS Grant No: 29137033). We further acknowledge institutional support from the School of Public Health, Faculty of Health Sciences, University of the Witwatersrand, South Africa; Centre de Recherche en Démographie, Université Catholique de Louvain, Louvain-la-Neuve, Belgium; and the African Population and Health Research Centre, Nairobi, Kenya, as critical bases for the MADIMAH project leadership. We gratefully acknowledge the South African Medical Research Council (SAMRC) for funding Carren Ginsburg’s Career Development Award.

Table of contents

[1 Introduction 5](#_Toc516039196)

[2 Computing hazard rates by age or by calendar time 5](#_Toc516039197)

[2.1 Hazard rates by age 6](#_Toc516039198)

[2.2 Hazard rates by calendar time 7](#_Toc516039199)

[3 Computing calendar rates by age group 7](#_Toc516039200)

[3.1 Preparing the dataset 7](#_Toc516039201)

[3.1.1 Split time into calendar years 7](#_Toc516039202)

[3.1.2 Split time into age group 8](#_Toc516039203)

[3.1.3 Smoothed hazard rates by age and period 9](#_Toc516039204)

[3.2 Tables for single failure (e.g. mortality) 10](#_Toc516039205)

[3.3 Tables for repeatable events (migration, fertility) 11](#_Toc516039206)

[4 Descriptive longitudinal analysis of non-renewable events 11](#_Toc516039207)

[4.1 Single non-renewable event: Kaplan-Meier estimates 11](#_Toc516039208)

[4.2 Non-renewable competing risks (e.g. causes of death) 13](#_Toc516039209)

[4.2.1 Nelson-Aalen Function (NAF) or cumulative hazard rates 13](#_Toc516039210)

[4.2.2 Cumulative incidence function (CIF) 14](#_Toc516039211)

[5 Descriptive longitudinal analysis of renewable events 15](#_Toc516039212)

[6 Special case: in-migration analysis 15](#_Toc516039213)

[7 Event history regression analysis 17](#_Toc516039214)

[7.1 The Cox model: semi-parametric proportional hazards model 17](#_Toc516039215)

[7.2 The Fine and Gray model for competing risks 18](#_Toc516039216)

Event history glossary as used in this manual (in alphabetical order)

**Cause-specific density**: the product of the survival rate at time *t* and the hazard rate at time *t, t+h*.

**Censoring (right-)**: time from which the individual is no longer observed

**Characteristic**: variable denoting an attribute of an individual that does not change over time (e.g. sex, birth year)

**Competing risks**: the set of mutually exclusive categories of a risk (e.g. causes of death) that sum up to the overall risk (e.g. death)

**Core event**: events that change the residency status of the individual (such as: enumeration, birth, death, in-migration, out-migration, end of observation)

**Core residency file**: a standardised file format containing the core events for each individual in the study/surveillance population, each event being recorded as a single record

**Cumulative hazard rates**: the sum of the hazard rates

**Cumulative incidence function** (CIF): the probability that a cause-specific event has occurred before time *t*. It is the sum over *t* of the cause-specific densities

**Event history analysis** (EHA): statistical regression analysis technique that identifies the effects of fixed and time-varying covariates on the time of occurrence of an event in presence of censoring

**Hazard rate**: the probability of experiencing the event in a short time interval *t, t+h*

**Kaplan-Meier function**: the probability that an event has occurred before time *t*

**Longitudinal data** (or event history data): data on characteristics, changes of status, and events with corresponding dates pertaining to each individual

**Nelson-Aalen Function (NAF)**: see Cumulative hazard rates

**Renewable event**: an event that can be repeated more than once during observation time

**Repeatable event**: see Renewable event

**Residency episode file**: a file where each episode of residence is marked with a start event and an end event in a single record with their corresponding time points

**Retrospective data**: data derived from questions concerning past events or changes of status starting from birth or from another fixed point in time

**Status**: variable denoting an attribute of an individual that may change over time (e.g. education level, BMI)

**Survival analysis** (also named time-to-event analysis, failure time analysis, duration analysis): descriptive statistical analysis technique that examines the time of occurrence of an event in the presence of censoring

**Truncation**: time until which the occurrence of an event is not known

# Introduction

To guide the reader through the present manual we assume that the reader is already familiar with the concepts and formats presented in the previous Manual of Event History Data Management^[[1]](#footnote-1)^. In particular, a standardised core residency file is essential to conduct both descriptive and in-depth longitudinal analysis.

An important operational distinction is between single (or non-renewable) events and repeatable (or renewable) events. These are also called single failure versus multiple failures. The corresponding estimators are not the same. In particular, the techniques applicable to renewable events (e.g. Nelson-Aalen function) can be applied to non-renewable events but the techniques applicable to non-renewable events (e.g. Kaplan-Meier, Cumulative Incidence Function) cannot be applied to renewable events. However, rates (*_n_m_x_*), and smoothed hazard rates (a good approximation of *_n_q_x_* probabilities for small time intervals) are exceptions to that rule, on the condition that the unit time of analysis (usually the year) is larger than the unit time used to record events (preferably time in days or months, at the most).

Table 1: Applicability of EHA techniques to single and repeatable events

| **Single (or non-renewable) events** | **Repeatable (or renewable) events** |
| --- | --- |
| Kaplan-Meier function | ~ |
| Nelson-Aalen function | Nelson-Aalen function |
| Cumulative Incidence Function | ~ |
| rates (*_n_m_x_*) | rates (*_n_m_x_*)* |
| smoothed hazard rates | smoothed hazard rates* |
| Cox models | Cox models |
| Fine and Gray (competing risks) model | ~ |

* If unit time of analysis > unit time used to record events
~: Do not apply to repeatable events.

In some sections of the manual, we will be analysing causes of death (CoD), i.e. we assume a datasets includes a categorical variable representing causes of death. This is to deal with competing risks.

# Computing hazard rates by age or by calendar time

Hazard rates (a.k.a. instantaneous hazard rates) are the simplest, most straightforward estimates of all. They can be computed in all cases: renewable or non-renewable events, competing risks or non-competing risks. They form the basis of all analyses whether descriptive or not. Nevertheless some caution is needed as to the time precision for data collection and for description:

1. Hazard rates make sense when the time precision for data collection is small as compared to the time of analysis, typically precision to the day when one wants to produce yearly rates. A precision to the month in data collection is the maximum that can be tolerated to produce yearly estimates. When the precision is the same (say, in years) for recording events and for event analysis then the rates can no longer be called hazard rates because they cannot be interpreted to instantaneous hazard rates. In other words, the recording time is not close enough to continuous time. With large units of time such as calendar year or age in years, the assumption of linear distribution of events and censoring is generally too strong, and the proportion of censored observations higher. However higher precision makes sense only if there are enough individuals and events to be observed for each time unit of analysis. Very rare events or small samples will not be suitable for hazard rates representation.
2. Even when the time precision for recording events in the field is sufficiently small, the hazard rates are not represented as such, e.g. for each day. For ease of interpretation, hazard rates are scaled (e.g. annualised) and then smoothed using a kernel density function following the principle of a moving average. The shape of the smoothed hazard rates will depend a lot on the choice of parameters for this kernel density function. The “width” parameter defines the number of time units before and after a centre point that will be used to compute the hazard estimate for that centre point (the width is actually half of the window around the centre point). The computation is done at each time unit, i.e. the windows are overlapping. The larger the width the smoother the estimate, e.g. a width of 1 year (i.e. 2-year window) will give smoother estimates than a 0.5 width. Various functions (or “kernel density functions”) are available that give different weights to hazards left and right of the centre point according to their distance to that centre point. Epanechnikov smoothing function is the default in Stata but simpler functions (e.g. a rectangle option, a simple moving average with equal weights given to hazards left and right of the centre point) might reflect the data quality better. With day precision in HDSS data, the default width is usually too large and actual trends are often more easily detectable with the rectangle smoothing function. Also, the smoothed hazard rates cannot be estimated for time units less than the minimum observed time + width, or the maximum observed time – width (see examples below).

The smoothed hazard rates are essentially visualisation tools. Actual rates cannot easily be extracted from these smoothed hazards. This is why the curves in Figure 1 of the attached research note are meant for communication and for pedagogical purposes but not for the production of precise measures as tackled in section 3.

## Hazard rates by age

These are the smoothed hazard rates by age of the population subjected to the same conditions during the observation period, whatever the duration of this period. The rates are not attached to one birth cohort but to multiple birth cohorts. Because the results reflect quite closely the yearly rates in a life table for a synthetic cohort, this kind of smoothed hazards often accompany the tables of rates (see next section).

* Compute the censoring and datebeg variables, if not already available

sort IndividualId EventDate EventCode

capture drop censor_death

bysort IndividualId (EventDate EventCode): ///

gen censor_death=(EventCode==7) if residence==1

count if censor_death==1

capture drop datebeg

bysort IndividualId (EventDate EventCode): ///

gen double datebeg=cond(_n==1, DoB, EventDate[_n-1])

format datebeg %tc

lab var datebeg "Date of beginning"

* Declare data to be suitable for EHA using age as analysis time

* origin() defines the beginning of analysis time

* scale() transform the scale of analysis time from days to years

* Display the number of milliseconds in an average year:

display 365.25*24*60*60*1000

* result=31557600000

stset EventDate if residence==1, ///

id(IndividualId) failure(censor_death==1) ///

origin(time DoB) time0(datebeg) scale(31557600000)

sts graph, hazard ci tmax(80)

sts graph, hazard ci width(0.5) tmax(80)

sts graph, hazard ci width(0.5) tmax(80) kernel(rectangle)

sts graph, hazard ci width(0.5) tmax(80) ylog kernel(rectangle) ///

ylabel(.001 .002 .005 .01 .02 .05 .1) ///

yline(.001 .002 .005 .01 .02 .05 .1, lpattern(dot)) ///

xlabel(0 1 5 10 15 20(10)80) xline(0 1 5 10 15 20(10)80, lpattern(dot)) ///

xtitle("Age in years") title("Smoothed mortality hazard")

## Hazard rates by calendar time

The smoothed hazard rates can be produced using calendar time since 1 January 1960 instead of age as analysis time. These are also called period hazard rates (cf. Figure 1 of the attached research note). For mortality, when no selection by age is done, these are simply estimates of Crude Death Rates for the whole population. These rates identify overall trends in the event of interest, which is useful for internal fact checking (e.g. do the known epidemics reflect in the data?) and external consistency checks (e.g. comparison of HDSS and district or national data).

* Declare data to be suitable for EHA using calendar time as analysis time

stset EventDate if residence==1, ///

id(IndividualId) failure(censor_death==1) time0(datebeg) ///

scale(31557600000)

sts graph, hazard ci

sts graph, hazard ci width(0.2)

Finally, the x-axis has to be relabelled remembering that the analysis time is time since 1 January 1960:

sts graph, hazard ci width(0.2) kernel(rectangle) ///

xlab(30 "1990" 35 "1995" 40 "2000" 45 "2005" 50 "2010" 55 "2015")

The period hazards are best used when limited to specific age groups (see next section).

# Computing calendar rates by age group

## Preparing the dataset

Before any computation of calendar rates by age group can be done, the data file must be shaped in such a way that variables for years (or any other calendar scale: month, season, semester, 2-year, 5-year, etc.) are readily available. This can be achieved by the stsplit command in Stata. The procedure is outlined below.

### Split time into calendar years

The stset command is the same as the one to produce hazard rates by calendar time:

* Declare data to be suitable for EHA using calendar time as analysis time

stset EventDate if residence==1, ///

id(IndividualId) failure(censor_death==1) time0(datebeg)

Then, the stsplit command is used to create an artificial event corresponding to the 1^st^ January of each calendar year (here from 2003 to 2015):

capture drop period*

gen period=0

forval year=2003/2015 {

* to get the exact value for 1 January of each year

di %20.0g tc(01Jan`year' 00:00:00)

* create a new record for each 1st January

local split=tc(01Jan`year' 00:00:00)

stsplit period`year', at(`split')

recode period`year' (`split'=`year')

replace period=period`year' if period`year'==`year'

drop period`year'

}

compress

sort IndividualId EventDate

Because a new event (1^st^ January) was created, we need a corresponding code for EventCode, the variable identifying event:

by IndividualId: replace EventCode=30 if EventCode==EventCode[_n+1] ///

& period!=. & period!=period[_n+1] & IndividualId==IndividualId[_n+1]

label define eventlab 30 "Period", modify

lab val EventCode eventlab

The censoring variable is no longer valid after an stsplit, so it is very important to compute the censoring variable and datebeg again after each stplit:

by IndividualId: replace datebeg=cond(_n==1, DoB, EventDate[_n-1])

sort IndividualId EventDate EventCode

cap drop censor_death

gen censor_death=(EventCode==7) if residence==1

cap drop censor_CoD_category

gen censor_CoD_category=cond(censor_death==1,CoD_category,censor_death)

lab val censor_CoD_category Cause_Category

### Split time into age group

The data can also be split by age group. This is not essential since commands to produce rates have an option to compute these rates by age as analysis time. However creating age groups can be quite useful to limit the analysis to some specific age ranges (e.g. under-5, 15-49, 60+).

In this case the analysis time must be age:

* Declare data to be suitable for EHA using birth as origin time

* origin() defines the beginning of analysis time

* scale() transform the scale of analysis time to years

stset EventDate if residence==1, ///

id(IndividualId) failure(censor_death==1) ///

origin(time DoB) time0(datebeg) scale(31557600000)

Here, we show how to split age (observation time) at age 1, 5 and then in increments of 5 up to 70. These ages are not computed at the exact birthday (since not all calendar years have the same length) but approximated using mean number of days in a calendar year:

capture drop age_group

stsplit age_group, at(1,5(5)70)

label def age_group 0 "<1" 1 "1-4" 5 "5-9" 10 "10-14" 15 "15-19" 20 "20-24" ///

25 "25-29"30 "30-34" 35 "35-39" 40 "40-44" 45 "45-49" 50 "50-54" ///

55 "55-59" 60 "60-64" 65 "65-69" 70 "70+"

lab var age_group age_group

compress

sort IndividualId EventDate

Because a new event (change in age group) was created, we need a corresponding code for EventCode:

by IndividualId: replace EventCode=40 if EventCode==EventCode[_n+1] & ///

age_group!=. & age_group!=age_group[_n+1] & IndividualId==IndividualId[_n+1]

label define eventlab 40 "AGE", modify

lab val EventCode eventlab

As for the split by calendar year, the censoring variable and datebeg has to be computed again:

by IndividualId: replace datebeg=cond(_n==1, DoB, EventDate[_n-1])

sort IndividualId EventDate EventCode

cap drop censor_death

gen censor_death=(EventCode==7) if residence==1

cap drop censor_CoD_category

gen censor_CoD_category=cond(censor_death==1,CoD_category,censor_death)

lab val censor_CoD_category Cause_Category

### Smoothed hazard rates by age and period

Splitting time into age groups is useful to produce period hazards (see preceding section) by age group:

* Declare data to be suitable for EHA using calendar time as analysis time

* In this example observation starts on 1st Jan 2003

stset EventDate if residence==1, ///

id(IndividualId) failure(censor_death==1) time0(datebeg) ///

origin(time tc(01Jan2000 00:00:00)) ///

entry(time tc(01Jan2003 00:00:00)) ///

scale(31557600000)

Note the use of origin and entry options. The analysis time is expressed in years since 1^st^ January 2000 (origin time) but the analysis starts on 1^st^ January 2003 (entry time).

* for infants and children :

sts graph if age_group<5, by(age_group) ///

hazard ci width(0.3 0.3) ///

tmin(3) kernel(rectangle) ///

ylab(.02 "20" 0.04 "40" 0.06 "60" 0.08 "80" 0.1 "100") ///

xlab(5 "2005" 10 "2010" 15 "2015") ///

legend(lab(5 "Infant death 1q0") lab(6 "Child death 4q1")) ///

title("Trends in Infant and Child Mortality per 1000 PYAR")

(see Figure 1 in Research Note)

* for young adults aged 15-29:

sts graph if age_group>=15 & age_group<30, by(age_group) ///

hazard ci width(0.5 0.5 0.5) ///

tmin(3.1) tmax(15.9) kernel(rectangle) ///

ylab(.005 "5" 0.01 "10" 0.015 "15") ///

xlab(5 "2005" 10 "2010" 15 "2015") ///

legend(lab(7 "5q15") lab(8 "5q20") lab(9 "5q25")) ///

title("Trends in Young Adult Mortality per 1000 PYAR")

* for adults aged 30-44:

sts graph if age_group>=30 & age_group<45, ///

hazard ci width(0.3) ///

tmin(3.1) tmax(15.9) kernel(rectangle) ///

ylab(.005 "5" 0.01 "10" 0.015 "15" 0.02 "20" 0.025 "25") ///

xlab(5 "2005" 10 "2010" 15 "2015") ///

legend(lab(3 "15q30")) ///

title("Trends in Adult Mortality per 1000 PYAR")

* for older adults aged 45-59:

sts graph if age_group>=45 & age_group<60, ///

hazard ci width(0.3) ///

tmin(3.1) tmax(15.9) kernel(rectangle) ///

ylab(0.01 "10" 0.02 "20" 0.03 "30" 0.04 "40") ///

xlab(5 "2005" 10 "2010" 15 "2015") ///

legend(lab(3 "15q45")) ///

title("Trends in Older Adult Mortality per 1000 PYAR")

* for oldest adults aged 60+:

sts graph if age_group>=60, ///

hazard ci width(0.3) ///

tmin(3.1) tmax(15.9) kernel(rectangle) ///

ylab(0.01 "10" 0.02 "20" 0.03 "30" 0.04 "40" 0.05 "50" 0.06 "60") ///

xlab(5 "2005" 10 "2010" 15 "2015") ///

legend(lab(3 "*q60")) ///

title("Trends in Oldest Adult Mortality per 1000 PYAR")

Splitting time by both age group and year (or any other time interval) is useful to compare the evolution of hazards by age over time:

* Declare data to be suitable for EHA using age as analysis time

stset EventDate if residence==1, ///

id(IndividualId) failure(censor_death==1) time0(datebeg) ///

origin(time DoB) scale(31557600000)

The evolution of hazards by age can be produced but is difficult to read if there are many periods:

sts graph if period>0, ylog tmax(80) hazard kernel(rectangle) ///

width(3 3 3 3 3 3 3 3 3 3 3 3) by(period) legend(cols(4))

The graphs are more readable by large age groups and 5-year periods:

recode period (2003/2007=2003) (2008/2012=2008) ///

(2013/max=2013), gen(period5year)

sts graph if period>0, ylog tmax(80) hazard kernel(rectangle) ///

width(2 2 2) by(period5year) legend(cols(4)) ///

ylab(.001 "1" .005 "5" .01 "10" .05 "50") ///

legend(lab(1 "2003-2007") lab(2 "2008-2012") lab(3 "2013- ")) ///

title("Hazard rates by age per 1000 - Log scale")

* Evolution for children under 5:

sts graph if age_group<10 & period>0, ylog tmax(5) ///

hazard ci width(1) kernel(rectangle) by(period5year) ///

ylab(.001 "1" .005 "5" .01 "10" .05 "50" .1 "100") ///

legend(lab(7 "2003-2007") lab(8 "2008-2012") lab(9 "2013- ")) ///

legend(cols(3)) title("Hazard rates by age per 1000 - Log scale")

* Evolution for young adults aged 15 to 29:

sts graph if age_group>=10 & age_group<35 & period>0, tmin(15) tmax(30) ///

hazard ci width(2) kernel(rectangle) by(period5year) ///

ylab(.01 "10" .02 "20" .03 "30" .04 "40") ///

legend(lab(7 "2003-2007") lab(8 "2008-2012") lab(9 "2013- ")) ///

legend(cols(3)) title("Hazard rates by age per 1000")

* Evolution for young adults aged 30 to 44:

sts graph if age_group>=25 & age_group<50 & period>0, tmin(30) tmax(45) ///

hazard ci width(2) kernel(rectangle) by(period5year) ///

ylab(.01 "10" .02 "20" .03 "30" .04 "40") ///

legend(lab(7 "2003-2007") lab(8 "2008-2012") lab(9 "2013- ")) ///

legend(cols(3)) title("Hazard rates by age per 1000")

* Evolution for young adults aged 45 to 59:

sts graph if age_group>=40 & age_group<65 & period>0, tmin(45) tmax(60) ///

hazard ci width(2) kernel(rectangle) by(period5year) ///

ylab(.01 "10" .02 "20" .03 "30" .04 "40") ///

legend(lab(7 "2003-2007") lab(8 "2008-2012") lab(9 "2013- ")) ///

legend(cols(3)) title("Hazard rates by age per 1000")

* Evolution for older adults above 60:

sts graph if age_group>=55 & period>0, tmin(60) tmax(80) ///

hazard ci width(2) kernel(rectangle) by(period5year) ///

ylab(.02 "20" .03 "30" .04 "40" .05 "50" .06 "60" .07 "70") ///

legend(lab(7 "2003-2007") lab(8 "2008-2012") lab(9 "2013- ")) ///

legend(cols(3)) title("Hazard rates by age per 1000")

## Tables for single failure (e.g. mortality)

These tables, also called period life table, are tables of rates, *_n_m_x_*, with *x*, the age at the beginning of the interval, and *n*, the time interval. The rates are produced by simply dividing, for a particular period and age group, the number of events by the total person-years at risk. They are similar to rates computed with aggregates from register data or civil registration data using the mid-period population as the population at risk, except that longitudinal data estimate the total person-years at risk with higher accuracy.

sort IndividualId EventDate EventCode

stset EventDate if residence==1, id(IndividualId) failure(censor_death==1) ///

origin(time DoB) time0(datebeg) scale(31557600000)

* One table for the period starting 2002

stptime if period>2002, at(0 1 5(5)85) by(Sex) per(1000) dd(2)

(see Table 1 in Research Note)

*** One table by calendar year:

*** annualised rates per 1000 person-years by age and sex for each year

forval year=2003/2015 {

display "Death rate by age group in `year'"

stptime if period==`year', at(0 1 5(5)85 120) by(Sex) per(1000) dd(2)

}

*** Table: annualised neonatal mortality rates per 1000

forval year=2003/2015 {

display "Neonatal death rate in `year'"

stptime if period==`year', at(0 0.0766598220396988) by(Sex) per(1000) dd(2)

}

To note, the latter neonatal mortality rate per 1000 is an annualised rate. To obtain the neonatal rate as used in textbooks (comparable to the probability of dying between birth and 28^th^ day), one must multiply the above rate by (28/365.25), i.e. the fraction 28 days over the average number of days in a year.

## Tables for repeatable events (migration, fertility)

These tables are the same as tables for non-repeatable events except that occasionally the rates can exceed one (i.e. 1000 per 1000 PYAR) when the event occurs particularly frequently.

**** Create censoring variable for out-migration

**** (accounts for rare cases where OMG or EXT at last observation, _n==_N)

sort IndividualId EventDate EventCode

capture drop censor_out

qui by IndividualId : gen censor_out=(EventCode==4 | EventCode==5) ///

& (residence[_n+1]==0 | (residence[_n+1]==. & _n!=_N)) if residence==1

count if censor_out==1

* MULTIPLE FAILURES PER INDIVIDUAL

stset EventDate if residence==1, id(IndividualId) failure(censor_out==1) ///

origin(time DoB) time0(datebeg) exit(time .) scale(31557600000)

***output as a table of rates per 100 by age and sex for each year

forval year=2003/2015 {

display "Out-migration rates by age group in `year'"

stptime if period==`year', at(0(5)85) by(Sex) per(100) dd(2)

}

# Descriptive longitudinal analysis of non-renewable events

Descriptive analysis of longitudinal data consists essentially of producing life tables or extinction tables for the whole population or for a sub-population. These tables depict the proportion of the population who survive at each age for a synthetic cohort, i.e. a cohort of individuals that would have been subjected over their lifetimes to the same, observed period’s conditions. Extinction tables differ from previous tables of rates in that the risk in each age interval is computed as a probability of experiencing the event in the age interval conditional on being alive at the beginning of the age interval, or *_n_q_x_* with *x*, the age at the beginning of the interval, and *n*, the time interval. In other words, the risk set is the cohort population at the beginning of the age interval, while in the calculation of rates (*_n_m_x_*), the population is the number of person-years at risk over the age interval.

## Single non-renewable event: Kaplan-Meier estimates

With individual data, the most popular estimator for an extinction table is the Kaplan-Meier (KM) method. The KM estimates account for the right-censored individual, i.e. those who are at risk but who stopped being observed before experiencing the event. Statistical software now can handle left-censoring as well, i.e. those individuals who were not part of the risk set before some time point. Therefore KM estimates may be produced for any calendar period, like rates.

A typical non-renewable event is death. However the same KM technique can be applied to any 1^st^ event such as first marriage, first birth, first migration, etc. The data preparation is the same as for rates.

*** Table of Kaplan-Meier nq0 probabilities from birth to age x by sex for each year

* Note: the Kaplan-Meier table ends at 120 to account for the maximum recorded age

* i.e. the last age interval starting at 85-years-old is open

sts list if period>2002, survival at(0 1 5(5)85 120) by(Sex)

(see Table 1 in Research Note)

forval year=2003/2013 {

display "Death probability between birth and age x in `year'"

sts list if period==`year', failure at(0 1 5(5)85 120) by(Sex)

}

*** Table of neonatal mortality probabilities

forval year=2003/2013 {

display "Neonatal death probability in `year'"

sts list if period==`year', failure at(0 0.0766598220396988) by(Sex)

}

Some probabilities are interesting to compute for specific age groups. For example, adult premature mortality is defined as the probability of dying between age 15 and age 60, _45_q_15_ and is computed by adding a restriction for entry time at 15-years old as follows:

*** Table of premature adult mortality probabilities (45q15)

*** (this probability is found at the intersection between

*** the column for failure function and the line for time 15)

forval year=2003/2013 {

display "Probability to die between 15 and 60 in `year'"

sts list if period==`year', failure at(15 60) by(Sex)

}

The median age at death or other quartiles and their confidence intervals are derived from the KM estimates and obtained by:

stci, p(25) by(Sex)

stci, median by(Sex)

stci, p(75) by(Sex)

The life expectancy at birth (or mean age at death) and its corresponding confidence interval is also derived from the KM estimates and is computed as follows:

* Restriction to longest follow-up time

forval year=2003/2013 {

display "Life expectancy at birth (e0) in `year'"

stci if period==`year', rmean by(Sex)

}

* For the whole 2003-2015 period

stci if period>2002, rmean by(Sex)

or (without the confidence interval):

* Exponential extension of survival time to zero

forval year=2003/2013 {

display "Life expectancy at birth (e0) in `year'"

stci if period==`year', emean by(Sex)

}

Life expectancy at 15-years old, e15, and median age at death conditional on survival at 15 can be computed by adding a restriction for entry time at birth plus 15-years old as follows:

stset EventDate if residence==1, id(IndividualId) failure(censor_death==1) ///

origin(time DoB) entry(time DoB+15*31557600000) time0(datebeg) scale(31557600000)

forval year=2003/2013 {

display "Median expected age at death at 15 in `year'"

stci if period==`year', median by(Sex)

display "Life expectancy at 15 (e15) in `year'"

stci if period==`year', rmean by(Sex)

}

## Non-renewable competing risks (e.g. causes of death)

Non-renewable competing risks occur when a particular unique event of interest, with a unique date of event for each individual, can be subdivided into several mutually exclusive categories. This is the case with causes of death, but also first migration destinations, type of first birth delivery, etc. In all these cases the event is not renewable. The usual KM estimate does not apply here since it would assume the date of competing risks as censoring dates. In other words KM would assume independence of competing risks to one another, which is an unrealistic assumption in most cases. Two techniques have been used to describe potentially non-independent competing risks: Nelson-Aalen Function (NAF, or cumulative hazard rates) and cumulative incidence function (CIF).

### Nelson-Aalen Function (NAF) or cumulative hazard rates

This popular technique consists simply of cumulating the hazard rates along the unit time of data collection for each of the risk categories, e.g. causes of death (CoD):

cap drop censor_death

gen censor_death=(EventCode==7) if residence==1

cap drop censor_CoD

gen censor_CoD=cond(censor_death==0,0,CoD_category)

lab val censor_CoD Cause_Category

stset EventDate if residence==1, ///

id(IndividualId) failure(censor_CoD==1) ///

origin(time DoB) time0(datebeg) scale(31557600000)

*** One table by calendar year

*** Table of Nelson-Aalen cumulative hazards by age and sex for each year

forval year=2003/2015 {

display "Cumulated death hazards from cause 1 in `year'"

sts list if period==`year', cumhaz at(0 1 5(5)85) by(Sex)

}

stset EventDate if residence==1, ///

id(IndividualId) failure(censor_CoD==2) ///

origin(time DoB) time0(datebeg) scale(31557600000)

*** One table by calendar year

*** Table of Nelson-Aalen cumulative hazards by age and sex for each year

forval year=2003/2015 {

display "Cumulated death hazards from cause 2 in `year'"

sts list if period==`year', cumhaz at(0 1 5(5)85) by(Sex)

}

This is repeated for as many categories of cause of death (CoD) as recorded. However, this technique has an important drawback: the scale in which the cumulative hazard rates are computed is open, i.e. it has no direct interpretation. Only the slopes of the NAF for each category can be compared to one another. This scale issue makes the NAF inconvenient for communicating results.

### Cumulative incidence function (CIF)

Recently the cumulative incidence function has become more popular than the NAF, though it is not yet included as a standard Stata command. Beware, it is a much more computer intensive technique since it requires estimating incidence (growth of hazard rates) from one time unit to the next rather than the hazard rates themselves. The advantage of the CIF is that its scale is interpretable as a probability like the failure function (i.e. the inverse of the survival function) since it ranges between the values 0 and 1. Actually the sum of CIFs (e.g. for each causes of death) is the failure function for all risks (e.g. overall mortality). The procedure is the following (e.g. for 3 causes of death and by a variable defining 2 birth cohorts):

* To install Stata user programs

net install stcompet

net install levels7

stset EventDate if residence==1, ///

id(IndividualId) failure(censor_CoD==1) ///

origin(time DoB) time0(datebeg) scale(31557600000)

The following command takes several hours to run on our dataset:

stcompet CIF_mean=ci CIF_hi=hi CIF_lo=lo if Sex==1, ///

compet1(2) compet2(3) compet3(9) by(period5year)

These last two commands compute the CIF for the first cause of death (coded 1 in our dataset) but also for each of 2 other causes of death (coded 2 and 3) plus indeterminate (coded 9). The command stcompet can handle up to 6 competing risks. To graph the curves (with confidence intervals) corresponding to each competing risk, new variables must be created for each competing risk (we discarded the indeterminate cause of death coded 9):

forval CoD = 1/3 {

foreach est in mean hi lo {

foreach per in 2003 2008 2013 {

gen CIF_CoD`CoD'_`est'_per`per'= CIF_`est' ///

if censor_CoD==`CoD' & Sex==1 & period5year==`per'

}

}

}

Then a graph can be drawn for the three competing risks by cohort (e.g. for males in 2003-2007):

twoway rarea CIF_CoD1_hi_per2003 CIF_CoD1_lo_per2003 _t ///

if Sex==1 & period5year==2003, sort color(gs14) ///

|| rarea CIF_CoD2_hi_per2003 CIF_CoD2_lo_per2003 _t ///

if Sex==1 & period5year==2003, sort color(gs14) ///

|| rarea CIF_CoD3_hi_per2003 CIF_CoD3_lo_per2003 _t ///

if Sex==1 & period5year==2003, sort color(gs14) ///

|| connected CIF_CoD1_mean_per2003 CIF_CoD2_mean_per2003 CIF_CoD3_mean_per2003 _t ///

if Sex==1 & period5year==2003, sort msymbol(none none none) legend(cols(3) rows(2)) ///

legend(lab(1 "95% CI") lab(2 "95% CI") lab(3 "95% CI") ///

lab(4 "AIDS/TB") lab(5 "Other") lab(6 "External")) ///

title("Competing death risks CIF for males in 2003-2007")

(see Figure 2 in Research Note)

# Descriptive longitudinal analysis of renewable events

All event history analyses of renewable events start with defining the event and within a longitudinal setting (here for out-migration analysis by age):

**** Create censoring variable for out-migration

**** (accounts for rare cases with OMG or EXT at last observation, _n==_N)

sort IndividualId EventDate EventCode

capture drop censor_out

qui by IndividualId : gen censor_out=(EventCode==4 | EventCode==5) ///

& (residence[_n+1]==0 | (residence[_n+1]==. & _n!=_N)) ///

if residence==1

stset EventDate if residence==1, ///

id(IndividualId) failure(censor_out==1) ///

origin(time DoB) time0(datebeg) scale(31557600000)

To our knowledge, there is currently no specific descriptive tool for renewable (repeatable) events. The suitable tools for analysing renewable events are borrowed from previous sections and are:

- The hazard rates: the smoothed hazard rates may exceed 1 when the risk of the renewable event is very high, i.e. when the event happens more than once on average per unit time (see section 2)
- The cumulative hazard rates (Nelson-Aalen Function) (see section 4.2.1)

It is worth noting that the CIF for competing risks cannot apply to renewable events.

# Special case: in-migration analysis

Everyone will agree that time goes in one direction and causes should precede effects. For example, all that contributes to death should be measured before death. What happens after the occurrence of a non-renewable event is irrelevant.

For renewable events, this is not the case since the individual outlives the event. Say that tertiary education measured before migration from place A to place B is contributing positively to migration out of A. What is answered through event history analysis is the question: at time t in place A, what are the chances of a tertiary-educated person experiencing an out-migration? Education or any characteristics of the migrant in B after migration might be the same or different from characteristics before migration, but in both cases they cannot be included as determinants of migration from place A to B.

However, this migration from A to B can be viewed from the place B of destination to answer the question: at time t in place B, what are the chances of a tertiary-educated person to have in-migrated? To answer this question with event history analysis, the analysis time must be… reversed!

Let us begin with the simplest case: producing rates by calendar time, say from 1^st^ January 2014 backward. Two new variables must be created:

**** Compute time backward from 1Jan2014

display %20.0f clock("1Jan2014","DMY")

* = 1704153600000

gen rev_EventDate1Jan2014=-(EventDate-1704153600000)

gen rev_datebeg1Jan2014=-(datebeg-1704153600000)

The censoring variable for in-migration must be created using reverse time:

sort IndividualId rev_datebeg rev_EventD

by IndividualId : gen censor_in=(residence[_n+1]==0 & EventCode[_n+1]!=4 ///

& EventCode[_n+1]!=1 & EventCode[_n+1]!=2) if residence==1

The stset command is executed to analyse data using time backward from 1st Jan 2014 as analysis time:

stset rev_datebeg1Jan2014 if residence==1, ///

id(IndividualId) failure(censor_in==1) ///

time0(rev_EventDate1Jan2014) exit(time .) scale(31557600000)

Then, the commands used for in-migration are the same as for repeatable events. For example, to get in-migration rates by period:

***output as a table of rates per 100 by age and sex for each year

stptime, at(0(1)10) by(Sex) per(100) dd(2)

Beware that in the resulting table the values 0 to 10 correspond to the year 2013, 2012, 2011, etc. backward to 2002 or less (labelled >10). Equivalently, one can use the variable period (assuming that data are already split by calendar year):

stptime, by(period) per(100) dd(2)

For in-migration analysis using age as analysis time, one must use reverse age. To do this one must decide on a maximum age beyond which in-migration cannot be measured reliably, say 70-year old (beyond that age out-migration rates are not reliable either due to small numbers):

****Compute age in reverse from the 70th birthday

capture drop rev_age*

gen rev_ageday70=(DoB+(70*365.25*24*60*60*1000))-EventDate

gen rev_agebeg70=(DoB+(70*365.25*24*60*60*1000))-datebeg

The stset command is executed to analyse the data using analysis time as the time going backwards from a 70^th^ birthday:

stset rev_agebeg70 if residence==1, ///

id(IndividualId) failure(censor_in==1) ///

time0(rev_ageday70) exit(time .) scale(31557600000)

Again, the commands used for repeatable events are suitable. For example, to get in-migration rates by age and by period (assuming that data are already split by calendar year):

***output as a table of rates per 100 by age and sex for each year

forval year=2003/2013 {

stptime if period==`year', at(0(5)70) by(Sex) per(100) dd(2)

}

As before the analysis time has to be interpreted in reverse to get the correct age. In the resulting tables the values 0 to 70 correspond to the age 70, 65, 60, 55, etc. backwards to 0 (i.e. date of birth). Also in an in-migration hazard curve by age, one must reverse and relabel the x-scale for better interpretation:

sts graph, hazard kernel(rectangle) width(.5 .5) xline(0(5)70) ///

xlab(70 "0" 60 "10" 50 "20" 40 "30" 30 "40" 20 "50" 10 "60" 0 "70") ///

tmax(70) xscale(reverse) cih by(Sex)

# Event history regression analysis

Once the steps for descriptive analysis are completed, the next steps for regression analysis are surprisingly easy. Essentially, all that is needed is a relevant stset command. The rest follows the standard regression literature.

## The Cox model: semi-parametric proportional hazards model

In event history analysis, the mother of all regression models is the semi-parametric proportional hazards model, a.k.a the Cox model, named after its inventor, David R. Cox. The basic principle of this model is to combine descriptive event analysis (the hazard rates) with regression analysis. The model belongs to the class of non-linear models since the dependent variable is log-transformed.

Log-transformation is generally needed with binary outcomes as is the case of the popular logit model. Actually a related model, the “discrete-time logit model”, mimics the Cox model, which is meant for (approximately) continuous time observations. There is common misconception that the discrete-time logit model is easier to handle than the Cox model. However, with data that have precision to the day as with HDSS data, it is absolutely counter-productive to use discrete-time logit model (e.g. using years as discrete time intervals) instead of a Cox model. Besides, discrete-time data are not easy to produce and discrete-time analysis not much easier to handle. The results would be comparable but the loss in precision would be considerable, leading to unnecessary biases due to added linear hypotheses and to higher event-censoring ties. In addition, analysis time (e.g. age) has to be controlled through a specific covariate in discrete-time logit model. Analysis time is therefore parameterised in this model, which presents an added constraint (the time intervals have to be carefully chosen).

Other popular models offer alternatives to the Cox model and to the discrete-time logit model, such as fully parametric models where hazard rates are constrained to follow a parametric function over analysis time (constant, Weibull, exponential, etc.). These are useful models in natural sciences but assumptions underlying parametric functions are more rarely applicable to human behaviour even with respect to biological process such as death. However, for specific age groups, it might be useful to use parametric models since they offer some flexibility to handle multilevel effects, informative censoring, etc.

Because this manual is but an introduction to event history analysis, only the Cox model and its competing-risks version (the next sub-section) will be presented here. As always, the analysis has a longitudinal data setting, say with age as analysis time. The procedure is the same for renewable and non-renewable events. Here we consider an analysis of out-migration:

**** Create censoring variable for out-migration

sort IndividualId EventDate EventCode

capture drop censor_out

qui by IndividualId : gen censor_out=(EventCode==4 | EventCode==5) ///

& (residence[_n+1]==0 | (residence[_n+1]==. & _n!=_N)) if residence==1

stset EventDate if residence==1, ///

id(IndividualId) failure(censor_out==1) ///

origin(time DoB) time0(datebeg) scale(31557600000)

To run a Cox model, one only needs to specify covariates and possibly some options:

* With sex and period as covariates, analysis limited to under-5 children

* Covariates are assumed to have independent effects

stcox i.period i.Sex if age_group<5

* With period as a covariate and sex as a stratification variable

* (the baseline hazard rates by age will be different for males and females

* and the covariates are assumed to have the same effect on both sexes)

stcox i.period if age_group<5, strata(Sex)

* Check that covariates have the same effect by running separate model by sex

stcox i.period if age_group<5 & Sex==1

stcox i.period if age_group<5 & Sex==2

When dealing with small samples or non-sample data (such as HDSS data, or subset of HDSS data), default standard errors computed using normality assumptions are not advisable as they may be sensitive to extreme values. Bootstrap standard errors are preferred as they are computed from the data only. Generally, the standard errors are larger with bootstrap standard errors and therefore the p-value are more conservative (meaning they show significance less often). Because bootstrap standard errors are based on replications (each replication is a regression model run on a sample of the data drawn with replacement) the procedure is quite computer-intensive. The number of replications by default is 50 but it is usually not sufficient to get asymptotic results. The minimum is 200 replications; the best is 500. Therefore, it is advised to first run a Cox model with default standard errors to check variable specifications and then to run the same model with bootstrap standard errors for the published version:

* With bootstrap standard errors (computer intensive)

stcox i.period if age_group<5, strata(Sex) vce(bootstrap, reps(200))

## The Fine and Gray model for competing risks

The Fine and Gray (F&G hereafter) model is, as the Cox model, a semi-parametric proportional hazards model. The difference is that the F&G model uses the Cumulative Incidence Function (CIF: see section on descriptive statistics) instead of the hazard rates used in the Cox model. This is to avoid making the assumption of independence between competing risks.

For example, when analysing mortality, increasing cancer may substitute declining cardiovascular disease. In other words, removing a cause of death may not translate in reduction of overall mortality because some other diseases may take over – a cause of death may hide another lurking behind it. When applying a Cox model to competing risks, the risk of interest becomes the dependent variable while the competing risks are treated as censored observations together with actual censored observations. Because censoring is assumed to be independent from the event, this assumption applies to competing risks in the Cox model, which might not be the case in reality. Instead, the F&G model treats competing risks distinctly from censoring.

The preparation of the data is the same as for non-competing risks:

* The following command is the same as for descriptive analysis of competing risks

stset EventDate if residence==1, ///

id(IndividualId) failure(censor_CoD==1) ///

origin(time DoB) time0(datebeg) scale(31557600000)

The variable censor_CoD is assumed here to take the value of 0 for censoring (no death), in addition to 1, 2, and 3 for three competing risks plus 9 for indeterminate cause of death.

The computation of a competing risk model using stcrreg (**s**urvival **t**ime **c**ompeting **r**isks **reg**ression) command is much more computer intensive than for the Cox model. Therefore it is advised to test the data with a Cox model and then to run the F&G model when everything else is in order before going to sleep, as the computation takes hours. The results are obtained for each competing risk in turn:

* To test data consistency:

stcox i.period i.Sex if age_group<5

* Note on coding: in this example of censor_CoD

* value 0 stands for ordinary censoring (no death yet),

* value 1 for the cause of interest, and

* values 2 and 3 for two competing causes of death

* value 9 for indeterminate (also competing but not analysed)

stcrreg i.period i.Sex if age_group>=15 & age_group<60, compete(censor_CoD==2 3 9)

* To obtain results for the two others causes:

stset EventDate if residence==1, ///

id(IndividualId) failure(censor_CoD==2) ///

origin(time DoB) time0(datebeg) scale(31557600000)

stcrreg i.period i.Sex if age_group>=15 & age_group<60, compete(censor_CoD==1 3 9)

stset EventDate if residence==1, ///

id(IndividualId) failure(censor_CoD==3) ///

origin(time DoB) time0(datebeg) scale(31557600000)

stcrreg i.period i.Sex if age_group>=15 & age_group<60, compete(censor_CoD==1 2 9)

Importantly, the F&G model more easily fails to converge if the number of events is too small. The rule of thumb is that a minimum of 150 events for the risk of interest (defined with the failure() option) is necessary for convergence. However, the competing risks (defined with the compete() option) may count less than 150 events.

Here also, bootstrap standard errors are advisable but the computation time is far larger than for the Cox model, literally days. The stcrreg command does not support multiprocessing options so it is not worth running on a supercomputer (trust us, we tried!). Your personal computer will seriously slow down while the stcrreg command is running, so it is best to run the bootstrap estimation on a devoted computer in batch mode. If you nevertheless want to use your own computer, you had better run the following command with bootstrap standard errors…:

* With bootstrap standard errors (very computer intensive)

stcrreg i.period i.Sex if age_group>=15 & age_group<60, ///

compete(censor_CoD==1 2 9) vce(bootstrap, reps(200))

… before going away on vacation!

1. Bocquier, P., Ginsburg, C., Herbst. K., Sankoh, O., Collinson, M.A. (2017). A training manual for Event History Data Management using Health and Demographic Surveillance System data. BMC Research Notes. 10: 224. [↑](#footnote-ref-1)
